# Radiogenomics in Colorectal Cancer

**DOI:** 10.3390/cancers13050973

**Published:** 2021-02-26

**Authors:** Bogdan Badic, Florent Tixier, Catherine Cheze Le Rest, Mathieu Hatt, Dimitris Visvikis

**Affiliations:** 1 National Institute of Health and Medical Research, LaTIM—Laboratory of Medical Information Processing (INSERM LaTIM), UMR 1101, Université Bretagne Occidentale, 29238 Brest, France; florent.tixier@univ-brest.fr (F.T.); catherine.cheze-le-rest@chu-poitiers.fr (C.C.L.R.); hatt@univ-brest.fr (M.H.); visvikis@univ-brest.fr (D.V.); 2 Department of Nuclear Medicine, University Hospital of Poitiers, 86021 Poitiers, France

**Keywords:** radiogenomics, machine learning, deep learning, oncologic imaging, genomics

## Abstract

**Simple Summary:**

Colorectal carcinoma is characterized by intratumoral heterogeneity that can be assessed by radiogenomics. Radiomics, high-throughput quantitative data extracted from medical imaging, combined with molecular analysis, through genomic and transcriptomic data, is expected to lead to significant advances in personalized medicine. However, a radiogenomics approach in colorectal cancer is still in its early stages and many problems remain to be solved. Here we review the progress and challenges in this field at its current stage, as well as future developments.

**Abstract:**

The steady improvement of high-throughput technologies greatly facilitates the implementation of personalized precision medicine. Characterization of tumor heterogeneity through image-derived features—radiomics and genetic profile modifications—genomics, is a rapidly evolving field known as radiogenomics. Various radiogenomics studies have been dedicated to colorectal cancer so far, highlighting the potential of these approaches to enhance clinical decision-making. In this review, a general outline of colorectal radiogenomics literature is provided, discussing the current limitations and suggested further developments.

## 1. Introduction

Worldwide, with over 1.8 million new colorectal cancer cases and 881,000 deaths, colorectal cancer ranks third in terms of incidence (10.2%) and second in terms of mortality (9.2%) [1]. Colorectal cancer (CRC) is a heterogeneous disease in terms of etiology, biology, therapy response, and prognosis. Environmental factors (e.g., high intake of red and processed meats, sugars, highly refined grains, smoking and heavy alcohol use, obesity), personal history of cancer, chronic inflammatory bowel diseases and age are well known examples of factors related to the increased risk of CRC [2]. Colorectal adenocarcinomas are the result of a stepwise progression from normal tissue epithelium to carcinoma. TNM classification (American Joint Committee on Cancer and the Union for International Cancer Control; TNM classification (T-Tumor; N-Node; M-Metastais), the most used classification for CRC, helps stratify patients into different therapeutic and prognostic subgroups), the most used classification for CRC, helps stratify patients into different therapeutic and prognostic subgroups [3]. The 5-year relative survival rate for CRC ranges from 90% in patients diagnosed with stage 1 disease to 70% for patients with regional spread, and down to 10–12% for patients with stage IV disease [4,5]. Within the same TNM stage, survival rates remain highly variable for different patients, which can be explained in part by tumor biological heterogeneity. Molecular biology showed that multiple events involving tumor-suppressor genes, oncogenes, and DNA mismatch repair genes contribute to the development of CRC, and the order in which these mutations occur is critical [6]. This results in spatial tumor heterogeneity (different tumoral clones within the primary tumor), intertumoral heterogeneity (tumoral heterogeneity between patients with the same histologic type), and temporal heterogeneity(differences that developed inside the tumor over time) [7].

Recently, tumor heterogeneity has also been assessed by radiomic features, i.e., quantitative metrics extracted from medical images. Different studies have investigated the prognostic value of radiomic features of patients with colorectal cancers derived from different modalities (e.g., PET, CT and MRI). Radiogenomics investigate the relationship between imaging features and gene expression alterations, and/or their potential added or complementary value in predictive oncological modeling.

The purpose of the present review is to provide a focused update on the rapidly evolving field of colorectal radiogenomics. We describe the workflow of colorectal cancer radiogenomics, their clinical application in oncology, and the future challenges in this field.

## 2. Radiomics

### 2.1. Radiomics Workflow

Medical imaging plays a central role in the detection, diagnosis, staging, and treatment of cancer and can provide morphological, structural, metabolic, and functional information [8]. The use of robust machine-learning techniques allows the extraction and exploitation of high-dimensional mineable data (morphologic, intensity-based, fractal-based, and textural features) from medical images for a more exhaustive characterization of tumor phenotypes [9]. The complexity of the radiomics workflow increases the need for standardized nomenclature and computation methods, which is being addressed by the Image Biomarker Standardization Initiative (IBSI). The IBSI currently provides standardized image biomarker nomenclature and definitions, a standardized general image processing workflow, tools for verifying radiomics software implementations, and reporting guidelines for radiomic studies [10]. Radiomics quality score (RQS) offers also a tool to analyze the methodology, reproducibility, and clinical applicability of radiomics studies [11].

Different modalities of medical imaging (CT [12], MRI, PET) are utilized for radiomics and radiogenomics studies. Before feature extraction can be carried out, the definition of a region/volume of interest (ROI/VOI) must be realized. It is one of the most challenging tasks in medical image analysis, especially in colorectal cancer, due to the size and shape of the organ and lesions (in any modality), as well as limited contrast between tissues (mostly in CT). The segmentation techniques of medical images are often specifically optimized for each combination of application, imaging modality, and body part [13]. In CT, because of the small intensity differences between primary colorectal tumor and adjacent tissues, the majority of studies relied upon a manual or semi-automated segmentation [14]. There are advantages and disadvantages to each approach. Manual segmentation requires expert annotation and is tedious, time-consuming, and prone to inter- and intra-observer variability. Semi-automatic segmentation methods have higher repeatability but may not always be as accurate as the manual segmentation in some situations (e.g., delineating the rectal tumor after neoadjuvant therapy) [15]. Automated segmentation is also subject to (more limited) variability. Therefore, the robustness of the radiomic features issued from multiple segmentations should be assessed in the workflow [16]. Fully automated segmentation methods, supervised and unsupervised, are generally built on basic image processing of pixel intensities and/or textural features, with the most promising methods relying on deep learning by training a U-net type structure [17]. Supervised techniques are considered to be more accurate but interobserver variability will still be present, as the manual part of the segmentation and the settings of the algorithm influence the result [12,18]. Unsupervised segmentation techniques commonly rely on labeled atlases and have been shown to be less accurate than the supervised techniques [19].

### 2.2. Features Extraction

Quantitative imaging features are subsequently extracted from the previously identified VOI. The features are usually classified into a number of families, such as intensity histogram-based features, shape-based features, texture-based features [20]. Several features require additional image processing steps before feature calculation (e.g., some intensity histogram metrics and all textural features require a prior discretization of intensities into a determined number of bins) [10].

### 2.3. Radiomics in Colorectal Cancer

Statistical analysis and prognostic model building are the next steps in radiomics analysis. Because a large number of radiomic features can be extracted from the image datasets, most of these exhibit intercorrelation and therefore are redundant. Combined with a small sample (i.e., number of patients), this can often contribute to the over-fitting of the models [21]. Feature selection should be performed to identify the most relevant and non-redundant subset of features that will be exploited to train a multiparametric prediction model according to the clinical endpoint [22]. Advanced machine and deep learning algorithms are employed for training/validation and testing of radio(geno)mics models. Various machine learning algorithms can be utilized—multivariate regression with least absolute shrinkage and selection operator (LASSO). LASSO serves as a regularization and variable selection method for any statistical model by shrinking the regression coefficients, and reducing some of them to zero [23]. Random forests (RF) is a machine learning algorithm that combines the predictions of a large number of small decision trees to produce a more accurate prediction [24]. Support vector machine (SVM) is an algorithm that performs classification by finding the hyperplane that maximizes the margin separating the classes [25].

Different studies have investigated the prognostic role of radiomics analysis in CRC. Radiomic analysis of colorectal cancer showed correlations with lymph node metastasis [26] and perineural invasion [27] or prediction of lung metastasis [28]. For colorectal liver metastasis, radiomics showed promising performance in predicting overall survival [29], disease-free survival [30], response to targeted therapies [31], the outcome for patients with unresectable hepatic metastases [32], or recurrence after liver resection [33]. In rectal cancer, radiomics were shown to be correlated with tumor regression [34], lymph node metastasis [35,36] or response after chemoradiation therapy [37,38].

## 3. Genomics and Transcriptomics

The development of CRC, from adenoma to adenocarcinoma, is the result of accumulated mutations in multiple genes that regulate cell growth and differentiation [39]. Colorectal carcinogenesis can arise from one or a combination of three different conditions, namely chromosomal instability (CIN), CpG island methylator phenotype (CIMP), and microsatellite instability (MSI) [40]. The classical CIN, observed in 65–70% of sporadic colorectal cancers, is associated with the acquisition of mutations in the adenomatous polyposis coli (APC), mutation of the *KRAS* oncogene, loss of chromosome 18q and deletion of chromosome 17p, which contains the important tumor suppressor gene *TP53* [41]. The consequence of CIN is an imbalance in chromosome number (aneuploidy), subchromosomal genomic amplifications, and a high frequency of loss of heterozygosity (LOH) [42]. CIN + CRCs are correlated with poorer survival irrespective of ethnic background, anatomical location, and treatment with 5-FU [43].

CpG island methylator phenotype CRC accounts for 15–20% of sporadic CRC and characterized by the vast hypermethylation of promoter CpG island sites, resulting in concomitant hypermethylation of multiple genes, silencing of normal tumor-suppressor function and cancer formation [44]. Many genes that have been identified to be affected in CIMP have important functions in the cell, (e.g., *CACNA1G*, *IGF2*, *NEUROG1*, *SOCS1* and *RUNX3*) [45]. CIMP-high CRCs are associated with distinct clinicopathological and molecular features such as older age, female preponderance, proximal tumor location, higher grade, reduced COX-2 expression, increased frequency of *TGFBR2* mutations, and high rate of *MSI*, *KRAS*, and *BRAF* mutations [46].

Microsatellite instability (MSI), detected in about 15% of all colorectal cancers, is a molecular phenotype due to a defective DNA mismatch repair system [47]. The MSI-high phenotype is characterized by mucinous or signet ring appearance, poor differentiation, proximal colon, prominent lymphocytic infiltration [48].

Transcriptomics is the study of the RNAs transcription and expression levels, functions, locations, trafficking, and degradation. RNAs studies allow the identification of genes that are differentially expressed in distinct cell populations or in response to different treatments [49]. The CRC Subtyping Consortium identified four molecular subtypes with distinguishing features: CMS1 (microsatellite instability immune, 14%), hypermutated, strong immune activation and microsatellite unstable; CMS2 (canonical, 37%), epithelial, marked WNT and MYC signaling activation; CMS3 (metabolic, 13%), epithelial and metabolic dysregulation; CMS4 (mesenchymal, 23%), prominent transforming growth factor–β activation, angiogenesis, and stromal invasion [50].

## 4. Radiogenomics in Colorectal Cancer

The molecular landscape of CRC has prognostic relevance and affects the choice of therapeutic strategies [51]. Carcinogenesis could be triggered by the activation of several pathways downstream the epidermal growth factor receptor (EGFR), (RAS/MAPK; SRC/FAK; PI3K/AKT pathway) through deregulation of protein synthesis, cell cycle, apoptosis, angiogenesis [52]. *KRAS* mutations are present in 30–50% of colorectal cancers and the RASCAL study of 2721 colorectal cancers showed that the presence of *KRAS* mutation was significantly associated with poorer prognosis [53]. K-Ras is a critical mediator of EGFR-induced signaling cascades and resistance to anti-EGFR therapies has been observed in patients with *KRAS* mutation [54]. Cetuximab and panitumumab, EGFR monoclonal antibody-based therapies that block ligand binding and lead to the inhibition of the downstream RAS-RAF-MEK-ERK signaling pathway, are reserved for patients with wild-type KRAS mCRC [55]. MSI-H non-metastatic CRC patients have improved survival and receive no benefit from fluorouracil (FU)-based adjuvant therapy [56]. Therapy with programmed cell death 1 (PD1)-blocking antibodies, pembrolizumab, and nivolumab have shown efficacy in patients with MSI metastatic CRC [57]. Other pathways implicated in initiation, progression, activation, and migration of CRC, such as Wnt/β-catenin, Notch, Hedgehog, and TGF-β/SMAD, (PI3K)/AKT could be potential sites for targeted therapy [58].

Since genomic analysis is now essential for therapy in colorectal cancer, there have been several attempts to explore a potential role of radiomics within this context, either as a surrogate of genomics (i.e., “virtual biopsy”) or as a complementary tool (i.e., added information). The development of radiogenomics models capable of predicting CRC genetic mutations is very useful in general practice to improve decision-making and patient outcomes.

### 4.1. ^18^F-FDG PET

The literature on CRC radiogenomics is limited but the rapidly increasing number of studies have examined the role of ^18^F-FDG PET in the radiogenomics assessment of *KRAS* mutations in CRC. Fluorodeoxyglucose positron-emission tomography/computed tomography (FDG PET/CT) allows for the evaluation of functional tumor activity via an assessment of metabolic activity and PET/CT is clinically indicated to evaluate for distant metastatic CRC patients.

In a study of 179 patients, Lee et al. found that the CRC patients with *KRAS* mutations had significantly higher standardized uptake value (SUV max) and SUV peak values than the patients expressing wild-type *KRAS* mutations [59]. A retrospective study of Kawada et al. found that *KRAS/BRAF* status could be predicted with an accuracy of 75% when a SUVmax cutoff value of 13 or 14 was used [60]. Interestingly these studies excluded patients with high C-reactive protein (CRP) levels because local inflammation and elevation in CRP could cause false-positive results in the ^18^F-FDG PET examinations [61]. Recently, Arslan et al. found that *KRAS* mutation and increased 2-FDG uptake are associated with a negative prognostic factor in CRC [62].

*KRAS*, *BRAF,* and *PIK3CA* mutations are highly concordant between primary tumors and distant metastatic CRC tumors, indicating that either type of tumor tissue could be useful as a source to detect *KRAS* mutations for the selection of anti-EGFR therapy [63,64]. For CRC metastases, the Krikelis et al. study including 58 stage IV patients found no correlation between SUV max and *KRAS* mutations, [65] Kawada et al., to minimize bias due to the partial volume effect, only considered tumors larger than 10 mm in diameter and showed that the KRAS mutation can be reliably predicted with an accuracy of 71.4% using a SUVmax threshold greater than 6.0 [66]. When considering only CRC liver metastasis Mao et al. reported SUVmax on both early and delayed scans predict patients harboring *KRAS* mutations [67].

Chen et al. found increased SUVmax and TW40% were associated in CRC tumors with *TP53* and *KRAS* mutations [68]. They also reported an association between contrast (gray-level co-occurrence matrix) and *KRAS* mutations, the TP53 mutation was associated with an increased value of short-run low gray-level emphasis (gray-level run length matrix) and APC mutations were correlated with lower low gray-level zone emphasis (gray-level zone length matrix). Miles et al. showed that *KRAS* mutations are associated with SUVmax, hypoxia-inducible factor-1 (HIF-1), and minichromosome maintenance protein 2 [69].

However, a number of studies have highlighted contradictory findings, concluding that none of the PET parameters could predict *KRAS* status in a meaningful way. In a study including 55 patients, Oner et al. cannot predict KRAS gene mutations using PET/CT parameters (SUVmax, metabolic tumor volume or total lesion glycolysis) [70]. Chen et al. in a study on 103 patients confirmed these results [68]. Krikelis et al. did not find any correlation between SUVmax measured in the metastasis and KRAS status analyzed in the primary cancer [65]. This can be partially be explained by the heterogeneity of the studied populations, SUVmax cut-off value, small sized tumors, etc. Table 1 summarizes the main PET/CT studies that have evaluated radiogenomics in colorectal cancer.

### 4.2. Magnetic Resonance Imaging

Magnetic resonance imaging (MRI) is the modality of choice for rectal cancer. The high soft-tissue contrast of MRI accurately assesses the extramural tumor spread and enables staging of both early and advanced rectal cancer, response assessment, and detection of recurrent disease [71]. Several studies evaluated the association between MRI derived features and *KRAS* mutations. Shin et al. found that tumors with *KRAS* mutations exhibited a longer axial length, as well as a larger ratio of the axial to the longitudinal dimensions on pretreatment rectal MRI [72]. With an accuracy of 0.640 (95% CI, 0.520 to 0.747, *p* = 0.0292), the study Jo et al. confirmed these findings [73]. Xu et al. reported that lower Max-ADC, Mean-ADC, pure diffusion, and higher pseudo-diffusion coefficient values were demonstrated in the *KRAS* mutant group [74]. They also reported that mean values of the six textural features (Mean, Variance, Skewness, Entropy, gray-level nonuniformity, run length nonuniformity) were significantly higher in *KRAS* mutant group compared to the *KRAS* wild type group (*p* < 0.0001) [75]. Histogram metric of diffusion kurtosis imaging showed moderate diagnostic significance for *KRAS* status in the Cui et al. study [76]. The same team found that a model combining seven radiomic features trained with support vector machine was able to predict the *KRAS* status with AUC of 0.722 (95% CI, 0.654–0.790) [77]. Meng et al. showed a radiomic model evaluating Ki-67 index with an AUC value of 0.699 (95% CI, 0.611–0.786) and *KRAS*-2 gene status with an AUC value of 0.651 (95% CI, 0.539–0.763) [78]. Hong et al. found no significant correlation between parameters of dynamic contrast-enhanced MRI and molecular prognostic factors (mutation of the *KRAS* oncogene status and microsatellite instability) [79]. Horvat et al. in a retrospective study of 65 patients found no correlation between radiomics and genetic mutations only significant correlation between qualitative MRI imaging features and genetic mutations [80]. Huang et al. model, built with clinical and radiomic features extracted from T2WI images, predicted the microsatellite instability status in rectal cancer [81]. Table 2 summarizes the main MRI studies that have evaluated radiogenomics in colorectal cancer.

### 4.3. CT SCAN

Computed tomography (CT) is the preferred imaging examination for colorectal cancer in clinical practice. Lubner et al. found that histogram skewness was negatively associated with *KRAS* mutation (*p* = 0.02) in patients with hepatic metastatic colorectal cancer [82]. He et al. investigated the predictive performance by using a residual neural network (ResNet) to estimate the *KRAS* mutation status on pre-treatment contrast-enhanced CT with an AUC 0.818 in testing cohorts [83]. Yang et al. reported an association between the CT texture signatures and the predicted *KRAS/NRAS/BRAF* mutations status of CRC when using a support vector machine model yielded an area under curve (AUC) of 0.869 (95% CI, 0.780–0.958) in the primary cohort and 0.829 (95% CI, 0.718–0.939) in the validation cohort [84]. Taguchi et al. showed that a multivariate support vector machine model including 14 CT textural features, yielded a significantly higher AUC (0.82) and thus a superior prediction performance when compared to the FDG PET-derived SUVmax for prediction of *KRAS* mutation status [85]. Dercle et al. retrospectively analyzed 667 patients from the randomized phase III multicenter trial CRYSTAL (NCT00154102) comparing fluorouracil, leucovorin, and irinotecan (FOLFIRI) plus cetuximab (cohort FC) with FOLFIRI alone (cohort F) in the first-line treatment of metastatic. The four radiomics features model, built using a Random Forest algorithm to diagnose anti-EGFR treatment-sensitive tumors, outperformed both *KRAS*-mutational status at baseline (AUC = 0.67, *p* < 0.001) and 8-week tumor shrinkage (RECIST 1.1-like unidimensional tumor shrinkage) [31]. Badic et al. showed correlations between the expression changes in four genes (*ABCC2*, *CD166*, *CDKNV1*, *INHBB*) and radiomic features in primary colorectal patients [86]. High *CXCL8* levels in CRC tumors are associated with poor prognosis in CRC patients [87] and Chu et al. showed correlations between CT radiomics features and CXCL8 expression [88].

Pernicka et al. combined two clinical variables and 40 radiomic features into a microsatellite instability (MSI) prediction model with an AUC 0.80 in training cohort and AUC of 0.79 in the validation cohort [89]. Fan et al. model predicting the MSI status combined six radiomic features and 11 clinical characteristics to achieve an AUC of 0.75 in stage 2 colorectal patients [90]. Wu et al. built a nine features model (three clinical features and six radiomic features) derived from dual energy computed tomography for predicting MSI status in CRC patients with an AUC of 0.961 in the training set and of 0.918 in the validation set [91]. Table 3 summarizes the main CT studies that have evaluated radiogenomics in colorectal cancer.

## 5. Limitations of Radiogenomics Studies

Despite the great potential of radiogenomics analysis in various oncologic applications, variability in feature extraction and lack of reproducibility are the main limitations of these studies [66,67]. Standardization of the imaging protocol with regard to dose administration, consistent acquisition parameters, and the use of reconstruction kernels with lower noise levels could be utilized in future radiomics studies [92]. In an analysis, of 41 studies including different imaging modalities and pathologies, Traverso et al. showed that first-order features were overall more reproducible than the shape metrics and textural features and entropy was one of the most stable first-order features [93]. Berenguer et al. found that only 71 of the 177 radiomic features extracted from CT images were reproducible and retained only 10 radiomic features because of redundant information in a two phantoms study to identify reproducible and non-redundant radiomic features for computed tomography [94].

Accurate identification and segmentation are essential for selectively gathering radiomic features from a tumor. Manual and semiautomated segmentation techniques are time-consuming and have high inter- and intrareader variability due to variability in the shape, size, poor contrast between adjacent organs and surrounding structures, organ position, etc., [95]. Utilization of automated segmentation approaches in abdominopelvic organs, such as detection of features, edges or intensities, clustering methods, strong or weak shape and/or location priors, thresholding and deformable models, could improve reproducibility of radiomic studies [96]. Standards for radiomic features are essentials for comparison of results between studies in the literature. An important initiative to advance the quality of radiomics research is the development of the publicly available Image Biomarker Standardization Initiative (IBSI) reference standard and associate recommendations that provide image biomarker nomenclature and definitions, benchmark data sets, and associated values enabling researcher to verify image processing and image biomarker calculations of their own software or implementation; reporting guidelines for high-throughput image analysis [10]. This can have an impact on radiogenomics studies as well, at least their radiomic component. 

Typically, thousands of quantitative imaging features can be extracted from images of tumors, in each available modality. Numerous genomics parameters can also be available. As a result, robust machine learning techniques for training reliable models have become mandatory. These methods have the ability to learn from data and thus automate and improve the prediction process, improve the performance of radiomics-based predictive models [97]. Parmar et al. valuated 14 feature selection methods and 12 classification methods in terms of their predictive performance and stability in NSCLC patients [98]. The authors emphasize the importance of selecting appropriate machine learning methods for each tumor type. However, too many features may include redundant and irrelevant information and cause overfitting. The number of features can be reduced before being considered as input to machine learning training, for example by performing test–retest analyses in patients or phantoms (to identify the most reliable/repeatable features) and by evaluating redundancy through correlation measurements [99].

Identification of the study population is important to avoid selection bias [100]. Variability exists in CRC incidence, age, race, prognosis, and risk factors [101]. In colorectal cancer *APC, KRAS, BRAF, PIK3CA* alterations are more common in older patients, and MSI-High, *TP53,* and *CTNNB1* mutations are more frequently observed in younger patients [102]. Histopathologic characteristics of tumors, node status, metastasis, microsatellite status, tumor grade, lymphovascular invasion molecular subtypes, characterization of the inflammatory infiltrate help stratify patients into different therapeutic and prognostic subgroups [103]. Microsatellite instability-high colorectal cancers have better overall survival and are associated with mucinous histology, a Crohn’s-like inflammatory response and, tumor-infiltrating lymphocytes [104]. Tumor infiltrating lymphocytes in CRC differs by primary tumor site; high CD81 cell density is associated with favorable prognostic factor for patients with right-sided colon tumors while CD31 cell density is a favorable prognostic factor for right colon and rectum tumors [105].

Heterogeneity exists in many morphological and physiological features, such as expression of cell surface receptors, proliferative, and angiogenic potential. Tumors often display intratumoral heterogeneity, i.e., several subpopulations within one tumor show differences in morphology, inflammatory infiltrate, mutational status, or gene expression profile [106]. Intratumor heterogeneity also takes place on a transcriptional level due to different cancer cells clone, tumor microenvironment, cell cycle, cell differentiation, and in response to local signaling [107]. Mutational timing, suggested by multiregional sequencing studies, showed that not only clone selection but also the timing of mutations contributes to the tumor heterogeneity and the relative clonal compositions of various regions of a developing tumor [108]. All these factors should be taken into consideration when building radiogenomics prognostic models, although these factors could end up being favorably replaced by more efficient radiogenomics. Different studies have already shown models combining clinical features with lower prognostic value than radiomics models [109], combined clinical and radiomic features models [89], or radiogenomics models [86].

Finally, the stability and reproducibility of the prognostic/predictive models should be assessed before applying them in a clinical setting [110]. Most studies have published results in relatively small datasets and internal validation may not be sufficient to extrapolate the performance in external datasets. Such external validation in a large multicenter setting is thus essential before implementing these prediction models in clinical practice [111].

## 6. Discussion and Future Directions

The use of radiogenomics for predicting correlations with genetic or transcriptomic tumor abnormalities in colorectal cancer patients still needs much larger data studies to fully validate the concept. A limitation of using “big data” in radiogenomics is that even though a lot of correlations may be derived using carefully controlled experiments, causality is notoriously difficult to establish [112]. Future research should focus on the limitations mentioned above, which remain the greatest barriers to adapting these tools for clinical application.

Multicenter, prospective clinical trials and creation of large and shared imaging/genomics datasets as a source of data that could be used by research teams around the world to develop and test new radiogenomics approaches, could be an interesting approach to advance the quality of radiogenomics studies and facilitate integration in clinical practice. Researchers should thus be encouraged to feed the existing databases (The Cancer Imaging Archive-TCIA, The Cancer Genome Atlas-TCGA, response to drug or radiation therapy -RIDER). Publicly available phantom datasets, intended for radiomics reproducibility tests, could help assess the influence of acquisition settings in order to eliminate non-robust radiomic features [113].

Relying on the radiomics quality score for assessment of homogeneous evaluation criteria and reporting guidelines could also contribute to improving the robustness and generalizability of future radiogenomics studies [112]. Finally, the transparent reporting of a multivariable prediction model for individual prognosis or diagnosis (TRIPOD) statement provides additional guidance for authors to report when developing or validating prediction models [114].

Intratumor or intermetastatic heterogeneity seems to affect drug response. The therapy strategy is decided on the basis of genetic evaluation from a small part of the primary tumor tissue, which might represent only a minor proportion of the subclones. Multiregion whole-exome sequencing revealed that metastatic tumors exhibit less intratumor heterogeneity than the primary tumors and inherit multiple genetically distinct subclones from primary tumors, supporting a possible polyclonal seeding mechanism for metastasis [115]. The correlations between these modifications and radiomics should be further evaluated.

## 7. Conclusions

In this review, we discussed the current utility of radiogenomics in colorectal cancer research, and described its potential future applications to clinical oncology. The ability to study biological phenomena with radiogenomics, added to conventional disease diagnosis and treatment, is expected to lead to significant advances in precision medicine. Further research in this rapidly evolving field is necessary in order to integrate radiogenomics in clinical decision-making.

## Figures and Tables

**Table 1:** Colorectal cancer radiogenomics studies based on PET/CT imaging.

Year	Author	Study	*N*	Study Population	Aim	Segmentation	Radiomic Features	Main Results	Internal Validation	External Validation	Conclusions
2020	Arslan	R	83	All stages	Prediction of the KRAS status	M	SUVmax	*KRAS* mutation mean SUVmax (24.0 ± 9.0); *KRAS* wild type mean SUVmax (17.7 ± 8.2)	N	N	Coexistence of *KRAS* mutation with higher SUVmax is a negative prognostic factor
2020	Popovic	R	37	Stage IV	Prediction of KRAS status in CRLM	M/A	SUV metrics corrected for tumor-to-blood standard uptake ratio (SUR) and partial volume effect (PVE)	SUV metrics(AUC 0.69–0.72); SUR metrics(AUC 0.73–0.75)	N	N	Corrected PET standard uptake values (SUV) correlated *KRAS* status
2019	Chen	R	74	All stages	Association between radiomics and genetic mutations	M	63 radiomic features	*KRAS* predictor histograms (OR 1.99) and contrast (OR 1.52) from GLCM predictors; SRLGE associated *TP53* (OR 243); LGZE predictor *APC* (OR < 0.001)	N	N	PET/CT-derived radiomics can determine KRAS, TP53, and APC genetic alterations
2018	Mao	R	49	Stage IV	Prediction of KRAS status in CRLM	M	Maximum standardized uptake value (SUVmax); change of SUVmax (DSUVmax); retention index (RI)	SUVearly AUC 0.694 (*p* = 0.002, 95% CI 0.582–0.807); SUVdelayed AUC 0.760 (*p* < 0.001, 95% CI 0.658–0.862); DSUVmax AUC 0.757 (*p* < 0.001, 95% CI 0.654–0.861); RI (%) AUC 0.684 (*p* = 0.003, 95% CI 0.571–0.797)	N	N	KRAS mutations predictors in CRLM: early and delayed SUVmax, DSUVmax, RI
2017	Oner	R	55	Na	Prediction of the KRAS status	A	SUVmax, SUVmean, MTV and TLG	SUVmax (AUC 0.54, OR 0.08, 95% CI, 0.38–0.7 *p* = 0.6); MTV (AUC 0.54, OR 0.08, 95% CI, 0.38–0.6 *p* = 0.6)	N	N	No significant association between KRAS gene mutations and SUVmax, MTV, TLG, NLR, PLR, CEA, CA 19-9 values
2016	Lee	P	179	All stages	Predict the KRAS status depending on C-reactive protein (CRP) levels	M/A	Maximum standardized uptake value (SUVmax), peak standardized uptake value (SUVpeak), metabolic tumor volume	None of the PET/CT-related parameters showed significant *KRAS* prediction; In normal CRP group, mutated *KRAS* associated with higher SUVmax (OR, 3.3; 95% CI, 1.4–7.4), SUVpeak (OR, 3.8; 95% CI, 1.5–9.3)	N	N	Higher SUVmax and SUVpeak values in *KRAS* mutated patients
2016	Lovinfosse	R	151	All stages	Prediction of *KRAS, NRAS, BRAF*	M	Standardized uptake values (SUVs), volume-based parameters and texture analysis	SUVcov highest AUC (0.65), sensitivity 56%, specificity 64%; SUVmax AUC 0.65 and sensibility 69% specificity 52%	N	N	The accuracy of ^18^F-FDG PET/CT quantitative metrics could not play a clinical role
2015	Chen	R	103	All stages	Prediction of TP53, KRAS, APC, BRAF, and PIK3CA	M	SUVmax, and various thresholds of metabolic tumor volume, total lesion glycolysis, and PET/CT-based tumor width (TW) were measured	SUVmax predicting *TP53*, OR 1.28 (95% CI, 1.01–1.61); TW 40% predicting *KRAS*, OR 1.15 (95% CI, 1.06–1.24)	N	N	Increased SUVmax and TW40% associated with TP53 and KRAS mutations
2015	Kawada	R	55	Stage IV	Prediction of the KRAS status	M	SUVmax	SUVmax (cutoff value 6.0) in tumors larger than 10 mm OR 0.78 (95% CI, 0.61–0.99) predicted *KRAS* status	N	N	^18^F-FDG accumulation into metastatic CRC was associated with *KRAS* status
2014	Chen	R	121	All stages	Prediction of the KRAS status	A	SUVmax; metabolic tumor volume, total lesion glycolysis, PET/CT-based tumor width	SUVmax OR 1.23 (95% CI, 1.01–1.52); TW 40% OR 1.15 (95% CI, 1.02–1.30).	N	N	SUVmax and TW40% were associated in CRC with KRAS mutations
2014	Krikelis	R	44	Stage IV	Prediction of the KRAS status	M	SUVmax	No correlation of SUVmax with KRAS status	N	N	No statistically significant correlation between SUVmax values and KRAS mutation status or GLUT1 mRNA levels.
2014	Miles	P	33	All stages	Prediction of the KRAS status	M	SUVmax, mean of positive pixels [MPP]), blood flow (BF)	The true-positive rate, false-positive rate, and accuracy (95% confidence intervals) of the decision tree were 82.4% (63.9%–93.9%), 0% (0%–10.4%), and 90.1% (79.2%–96.0%), respectively.	Y	N	Combined measurements of tumor ^18^F-FDG uptake, CT texture, and perfusion has the potential to identify KRAS mutations
2012	Kawada	R	51	All stages	KRAS/BRAF mutations affect FDG accumulation in CRC	M	Radiomic features	*KRAS* and *BRAF* mutations correlated with SUVmax (OR, 1.17; 95% CI, 1.03–1.33), TLR (OR, 1.40; 95% CI, 1.08–1.80)	N	N	FDG accumulation was higher in CRC with KRAS/BRAF mutations

Abbreviations: sens: sensitivity; spec: specificity; retrospective (R); prospective (P); not available (Na); area under the curve (AUC); microsatellite instability (MSI); area under curve (AUC); Kirsten rat sarcoma viral oncogene ( KRAS); *NRAS* Proto-Oncogene, GTPase (NRAS); (CR)—colorectal tumors, (R)—rectal tumors, (SRLGE)—short-run low gray-level emphasis; colorectal liver metastases (CRLM); maximum standardized uptake value (SUVmax); change of SUVmax (DSUVmax); retention index (RI); standard uptake ratio (SUR); partial volume effect (PVE); tumor-to-blood standard uptake ratio (SUR); mean of positive pixels (MPP); blood flow(BF); Na—not available, manual segmentation (M); manual/automatic segmentation (M/A).

**Table 2:** Colorectal cancer radiogenomics studies based on magnetic resonance imaging. All studies included rectal tumors.

Year	Author	Study	*N*	Study Population	Aim	Segmentation	Radiomic Features	Main Results	Internal Validation	External Validation	Conclusion
2020	Wang	R	306	Na	Deep model to independently predict the genetic status of KRAS mutations	M	DL model	MBCAM2 model- accuracy 90.50%, sens 92.79%, spec 87.64%, and AUC 96.00%	Y	N	Multi-branch cross attention model outperforms all the methods of DL
2020	Oh	R	60	All stages	Prediction of KRAS status	M	Three radiomic model	sens (84%), spec (80%), accuracy values (81.7%), AUC (0.884) of the decision tree for the whole dataset	Y	N	Three MRI imaging features that could predict KRAS status
2020	Cui	R	304 + 86	Na	Prediction of KRAS status	M	Seven radiomics features	Training dataset AUC of 0.722 (95% CI, 0.654–0.790); internal validation AUC 0.682 (95% CI, 0.569–0.794); external validation AUC 0.714 (95% CI, 0.602–0.827)	Y	Y	Moderate performance to predict KRAS status
2019	Horvat	R	65	Na	Correlations between genetic mutations and radiomics	M	Thirty-four texture features	No associations between clusters/qualitative features and gene mutations (except for PTPRT)	N	N	Associations between quantitative features and genetic mutations; pas de correlations between qualitative features and genetic mutations
2019	Cui	R	148	Exclusion Stage IV	Prediction of the status of KRAS	M	D, K, and apparent diffusion coefficient (ADC) values	K75th AUC value of 0.871 (0.806–0.920) sensitivity 81.43%, specificity78.21%, positive predictive value 77.03%, negative predictive value 82.43%	N	N	DKI metrics with whole-tumor volume histogram analysis is associated with KRAS mutation
2019	XU	R	158	Na	Prediction of KRAS status	M	Mean, Variance, Skewness, Entropy, gray-level nonuniformity, run length nonuniformity	texture features AUC (0.703–0.813); ADC values (AUC 0.682, 95% CI: 0.564–0.801), sensitivity (66.67) and specificity (62.12%)	N	N	Mean values (Mean, Variance, Skewness, Entropy, gray-level nonuniformity, run-length nonuniformity) higher in KRASmt group
2018	JO	R	75	Na	Prediction of KRAS status	M	Tumor length, ADC, relative contrast enhancement	The higher ratio of axial to LTL in the KRAS-mutant group AUC 0.640 (95% CI, 0.520 to 0.747, *p* = 0.0292), maximum accuracy of 64%	N	N	Ratio of axial to longitudinal tumor lengths predicted KRAS mutation (accuracy of 64%)
2018	XU	R	51	Na	Prediction of KRAS status	M	Max-ADC, Min-ADC, Mean-ADC, pure diffusion, perfusion fraction, pseudo-diffusion coefficient	Kras status AUC values of Max-ADC, Min- ADC, Mean-ADC, D, f and D* were 0.695, 0.604, 0.756, 0.701, 0.599 and 0.710	N	N	Lower Max-ADC, Mean-ADC and D and higher D values observed in the KRAS mutant group
2018	Meng	R	345	Na	Radiomic model’s prediction of biological characteristics	M	DL model	Model Ki-67 (AUC 0.699 95% CI, 0.611–0.786); HER-2 (AUC 0.696, 95% CI, 0.610–0.782) Ki-67; KRAS-2 (AUC0.651, 95% CI, 0.539–0.763),	Y	N	Radiomic signatures correlated to HER-2, KRAS-2 gene status
2016	Shin	R	275	All stages	Prediction of KRAS status	M	Axial tumor length, ratio of the axial to the longitudinal tumor dimensions	KRASm tumors- longer axial length, larger ratio of the axial to the longitudinal dimensions.	N	N	KRAS status associated with gross tumor pattern, axial length, ratio of the axial to the longitudinal dimensions of the tumor
2013	Hong	R	29	Na	correlations between parameters of dynamic contrast-enhanced magnetic resonance imaging and prognostic factors	M	Steepest slope (SLP), time to peak (Tp), relative enhancement during a rapid rise (Erise), maximal enhancement (Emax)	Erise was significantly correlated with N stage, and Tp was significantly correlated with histologic grade	N	N	no significant correlations between DCE-MRI parameters and K-ras mutation, microsatellite instability

Abbreviations: sens: sensitivity; spec: specificity; retrospective (R); manual (M); not available(Na); apparent diffusion coefficients (ADC); area under the curve (AUC); microsatellite instability (MSI); area under curve (AUC); Kirsten rat sarcoma viral oncogene (KRAS); *NRAS* Proto-Oncogene, GTPase (NRAS); B-Raf Proto-Oncogene (BRAF); standard deviation (SD); Steepest slope (SLP), time to peak (Tp); dynamic contrast-enhanced(DCE); diffusion-weighted imaging(DWI); relative enhancement during a rapid rise (Erise); maximal enhancement (Emax); magnetic resonance imaging (MRI). D*-Pseudo-diffusion coefficient.

**Table 3:** CT scan imaging radiogenomics studies of colorectal cancer published in literature.

Year	Author	Study	*N*	Study Population	Aim	Segmentation	Radiomic Features	Main Results	Internal Validation	External Validation	Conclusion
2020	HE	R	117 + 40	All	Predictive performance by using residual neural network (ResNet) to estimate the KRAS status	M	4 features radiomics model	Radiomics model training cohort, AUC 0.945 (sens: 0.75; spec: 0.94); testing cohort, AUC 0.818 (sens: 0.70; spec: 0.85). ResNet model AUC 0.90 testing cohort	Y	N	Better prediction of Kras status by residual neural network than radiomics model
2020	CHU	R	99 + 42	All	Relationship among prognosis, radiomics features, and gene expression	M	12 radiomics features	Radiomic model training cohort AUC 0.829 (95% CI: 0.750–0.908) testing cohort AUC 0.727 (95% CI: 0.570–0.884)	Y	N	Radiomics model reflected by CXCL8 combined with tumor stage information predict the prognosis
2020	Negreros-Osuna	R	145	Stage IV	Prediction of BRAF mutation	M	Standard deviation (SD), the mean value of positive pixels (MPP)	Lower SD 22.31 (95% CI: 20.66, 24.62) and MPP 51.54 (95% CI: 47.14, 58.99) in BRAF mutant tumors	N	N	Radiomics texture features predictors of BRAF mutation status and 5-year OS
2020	González-Castro	R	47	All	Prediction of KRAS status	M	Radiomic model (second-order features)	Neural Networks model sens of 88.9%, spec 75.0%, accuracy of 83%	Y	N	Prediction of KRAS status in CRC
2020	Dercle	R	667	Stage IV (CRYSTAL trial (NCT00154102))	Predict tumor sensitiveness to FOLFIRI ± cetuximab.	M	4 features radiomics model	AUC 0.80 (95% CI: 0.69–0.94) sens 0.80, and spec 0.78); *p* < 0.001)	Y	N	Performance of the signature outperformed both KRAS-mutational status at baseline
2019	Pernicka	R	139 + 59	Stage II and III	Prediction of (MSI) status	M	40 radiomic features	AUC of 0.80 for the training set and 0.79 for the test set (spec = 96.8% and 92.5%, respectively)	Y	N	The combined model performed slightly better than the other models
2019	Taguchi	R	40	Stage II-IV	Prediction of KRAS status	M	14 CT radiomics et SUV max	Multivariate support vector machine CT radiomics model AUC of 0.82 superior compared to the SUVmax.	N	N	CT texture analysis was superior to the SUVmax for predicting the KRAS mutation status
2019	Wu	R	102	Na	Prediction of (MSI) status	M	6 radiomics features	Training set AUC 0.961 (accuracy: 0.875; sens: 1.000; spec: 0.812); testing set AUC of 0.875 (accuracy: 0.788; sens: 0.909; spec: 0.727)	Y	N	Radiomics analysis of iodine-based material decomposition predict MSI status
2019	Fan	R	119	Stage II	Prediction of (MSI) status	Semiautomatic	6 radiomics features	Radiomic model AUC = 0.688; accuracy = 0.713; sens = 0.517; spec = 0.858; clinical model 0.598 AUC value, 0.632accuracy, 0.371 sens, and 0.825 spec; combined model AUC 0.752 (accuracy = 0.765; sens = 0.663; spec = 0.842).	N	N	Better detection of MSI status with combined clinical and radiomics feature model than clinical/radiomics alone
2019	Badic	R	64	All	Prognostic value of gene expression and radiomics	M	Shape, second and third order texture features	PFS Cox model combining Stage 3, ABCC2 and Entropy_GLMC_ HR 22.8 95% CI 3.7 to 141 *p* < 0.0001 OS Cox model with Ratio and ALDH1A HR 8.4 95% CI 3.4 to 20.6 *p* = 0.0005	N	N	Model combining CE-CT radiomics, gene expression, histopathological examination could provide higher prognostic stratification power
2018	YANG	R	61 + 56	All	Predict KRAS/NRAS/BRAF mutations	M	3 radiomics features	Testing cohort AUC 0.869, sens 0.757, and spec 0.833; Validation cohort AUC 0.829, sens 0.686, spec 0.857	Y	N	Prediction of KRAS/NRAS/BRAF mutations
2015	Lubner	R	77	Stage IV	CT texture features relate to pathologic features and clinical outcomes	M	First class radiomics	Skewness was negatively associated KRAS mutation (*p* = 0.02).	N	N	MPP, SD, correlates overall survival

Abbreviations: sens: sensitivity; spec: specificity; retrospective (R); manual (M); not available (Na); microsatellite instability (MSI); area under curve (AUC); Kirsten rat sarcoma viral oncogene (KRAS); *NRAS* Proto-Oncogene, GTPase (NRAS); B-Raf Proto-Oncogene (BRAF); standard deviation (SD); the mean value of positive pixels (MPP), computed tomography (CT).
